# Moracin attenuates LPS-induced inflammation in nucleus pulposus cells via Nrf2/HO-1 and NF-κB/TGF-β pathway

**DOI:** 10.1042/BSR20191673

**Published:** 2019-12-04

**Authors:** Ronghe Gu, Zonggui Huang, Huijiang Liu, Qiwen Qing, Zou Zhuan, Lijing Yang, Zhongyi Su, Weiguo Huang

**Affiliations:** Department of Orthopedics, The First People’s Hospital of Nanning, Nanning 530022, China

**Keywords:** inflammation, intervertebral disc degeneration, LPS, Moracin, Nrf-2

## Abstract

The present study was designed to investigate the protective effect of moracin on primary culture of nucleus pulposus cells in intervertebral disc and explore the underlying mechanism. Moracin treatment significantly inhibited the LPS-induced inflammatory cytokine accumulation (IL-1β, IL-6 and TNF-α) in nucleus pulposus cells. And moracin also dramatically decreased MDA activity, and increased the levels of SOD and CAT induced by LPS challenge. Moreover, the expressions of Nrf-2 and HO-1 were decreased and the protein levels of p-NF-κBp65, p-IκBα, p-smad-3 and TGF-β were increased by LPS challenge, which were significantly reversed after moracin treatments. Moracin treatments also decreased the levels of matrix degradation enzymes (MMP-3, MMP-13) as indicated by RT-PCR analysis. However, Nrf-2 knockdown abolished these protective effects of moracin. Together, our results demonstrated the ability of moracin to antagonize LPS-mediated inflammation in primary culture of nucleus pulposus in intervertebral disc by partly regulating the Nrf2/HO-1 and NF-κB/TGF-β pathway in nucleus pulposus cells.

## Introduction

Recently, growing evidences have shown that the mechanism of pain induced by degeneration of intervertebral disc is still not very clear, and the clinical efficacy is not good. Intervertebral disc degeneration, when comes to the condition of cell viability decrease, attenuation of type II collagen and proteoglycan synthesis and dehydration of nucleus pulposus, has been considered as an irreversible process [1]. Also, intervertebral disc degeneration is a significant contributor to the development of low back pain and other spinal degenerative diseases [2]. And now there is growing evidence that disc degeneration is a disease that is closely related to the nucleus pulposus cells inflammation [3,4]. Although the cause of disc degeneration is still ambiguous, inflammation and oxidative stress abnormalities are considered to be important causes of the disease.

Inflammation has been suggested to be closely involved in the pathological process of intervertebral disc degeneration [5]. The inflammatory cytokines inducing interleukin-1β (IL-1β), IL-6 and tissue necrosis factor-α (TNF-α) play an important role in intervertebral disc degeneration [6]. The elevated inflammatory factors are a stimulant of the inflammatory cascades. Therefore, inhibition of the release of inflammatory cytokines is a very effective and feasible solution in preventing degeneration of the intervertebral disc. [7].

Moracin is a biological active component obtained from the root barks of *Morus alba* L. (family Moraceae), which have been widely used in traditional medicine for the treatment of various inflammatory conditions in Asia [8]. Moracin was reported to inhibit airway inflammation by regulating the NF-κB and JNK/c-Jun signaling [9]. In lipopolysaccharide-activated microglia, moracin showed inhibitory activities against nitric oxide productions [8]. However, it has not been reported before on the effective role and its underlying mechanism of moracin in the intervertebral disc degeneration. The present study was designed to study the effects of moracin on LPS-induced primary culture of nucleus pulposus in intervertebral disc and explore the underlying mechanism through Nrf-2/HO-1 and NF-κB/TGF-β pathway.

## Materials and methods

### Reagents

The drug, moracin (wkq-00871) was purchased from Sichuan Victory Biological Technology Co., Ltd (Chengdu, China). LPS (Escherichia coli O111:B4) was purchased from Sigma (St. Louis, MO, U.S.A.). Catalase (CAT, LE-06378), malondialdehyde (MDA, LE-07345) and superoxide dismutase (SOD, LE-07334) detection kits were bought from Lai Er Bio-Tech (Hefei, China). Enzyme-linked immunosorbent assay (ELISA) kits for IL-6 (CRC0063), TNF-α (BMS622) and IL-1β (BMS630) were obtained from eBioscience. CO., LTD. All the primary antibodies used in the present study, including antibodies for Nrf-2 (ab137550), HO-1 (ab13243), TGF-β (ab190503), p-smad-3 (ab193297), smad-3 (ab40854), p-IκBα (ab133462), IκBα (ab32518), p-NF-κBp65 (ab86299), NF-κBp65 (ab16502) were from Abcam (Cambridge, U.K.).

### Nucleus pulposus cells isolation and culture

In the present study, the lumbar spines from Sprague Dawley rats were used to isolate nucleus pulposus cells. All animal procedures were approved by the Animal Care and Use Committees of the First People’s Hospital of Nanning and animal experiment was performed in the Animal Center of the First People’s Hospital of Nanning (Nanning, China). After separated and washed, the nucleus pulposus tissues were digested with trypsin and collagenase to get the nucleus pulposus cell. And the cell was maintained in Dulbecco’s modified Eagle’s medium (DMEM, Gibco BRL) containing 15% (v/v) fetal bovine serum (FBS, HyClone), with additional 100 U/ml penicillin, 100 mg/ml streptomycin (Invitrogen) at 37°C under 5% CO_2_. And in the present study, the concentration of LPS with 10 μg/ml was used to induce the inflammation.

### Cell viability assay

To evaluate the potential cytotoxic effects of moracin, cell viability assay was performed by Cell Counting Kit-8 (C008-3, CCK-8, 7sea biotech, Shanghai, China). The nucleus pulposus cells were plated in 96-well plates with the density at 5 × 10^3^ per well. The nucleus pulposus cells were incubated with moracin at concentrations (2.5, 5, 10, 20, 40,80, 160 μM) for 24 h, then 10 ml CCK-8 solution was added for incubation another 2 h. The optical density at 450 nm was used to measure the cell viabilities using a spectrophotometric plate reader (BioTek, U.S.A.).

### Small interfering RNAs, plasmids and transfection

The nucleus pulposus cells were plated on 6-well plates with 4 × 10 ^4^ cells/ml in 1-ml culture medium. Nrf-2 siRNA (#5285, Cell Signaling Technology) (forward 5′-GGAGAGCCCAAUGUUUCAUTT-3′ and reverse 5′-AUGAAACAUUGGGCUCUCCTT-3′) transfections were performed according to the manufacturer’s instructions of ExFectTM Transfection Reagent (Vazyme Biotech). Then, the cells were incubated at 37°C for 6 h and harvested for further experiments.

### Inflammatory cytokines measurement in cell supernatant

The concentrations of inflammatory cytokines IL-1β, IL-6, TNF-α in cell supernatant were recorded by an enzyme-linked immunosorbent assay (ELISA) kits according to the manufacturer’s recommendations (eBioscience Inc., San Diego, CA).

### SOD, MDA and CAT measurement in cell supernatant

The levels of superoxide dismutase (SOD), malondialdehyde (MDA) and catalase (CAT) in cell supernatant were recorded using the commercial kits on the basis of the manufacturer’s instruction.

### Real-time PCR

The nucleus pulposus cell was incubated with moracin for 2 h before stimulating by 10 μg/ml LPS. Briefly, the total RNA of the nucleus pulposus cells was extracted using TRIzol reagent (Invitrogen Co., U.S.A.). About 6 μl extracted RNA was reverse transcribed using the PrimeScript™ RT reagent Kit with gDNA Eraser (TAKARA) according to the provider’s protocol. Quantitative PCR was performed using SYBR® Green Real time PCR Master Mix (TAKARA) in the StepOnePlus Real-Time gliomaR System (ABI Prism 7500 fast). GAPDH was used as the internal reference. The Sequences of the primers for MMP-3, MMP-13, Col-I, Col-II and aggrecan used in the polymerase chain reaction as seen in Table 1.

**Table 1:** Sequences of the primers used in the polymerase chain reaction (PCR)

Gene	Primer sequences (5–3)
MMP-3	Forward: TTTGGCCGTCTCTTCCATCC
MMP-13	Forward: ACCATCCTGTGACTCTTGCG
Collagen I	Forward: ATCAGCCCAAACCCCAAGGAGA
Collagen II	Forward: TGATGGGATCCAATGAGGGAGA
Aggrecan	Forward: AGGGACACCAACGAGACCTA
GAPDH	Forward: CAGAAGGACAGCTACGTGGG

### Western blot

The cells were homogenized, washed with PBS and dissolved in RIPA buffer (Beyotime, Shanghai, China) and the quantitative protein concentration was determined by Enhanced BCA Protein Assay Kit (Beyotime, Shanghai, China). The total protein content was quantified and equal amounts of protein were loaded on 8–12% SDS-polyacrylamide gel electrophoresis (Mini-Protean-3, Bio-Rad, Hercules, CA, U.S.A.) and transferred onto a PVDF transfer membrane (Millipore, Massachusetts, U.S.A.). After blocking the membrane, they were incubated with specific primary antibodies, Nrf-2 (1:1000, ab137550), HO-1 (1:1000, ab13243), TGF-β (1:1000, ab190503), p-smad-3 (1:1000, ab193297), smad-3 (1:1000, ab40854), p-IκBα (1:1000, ab133462), IκBα (1:1000, ab32518), p-NF-κBp65 (1:1000, ab86299), NF-κBp65 (1:1000, ab16502), overnight at 4°C followed by further incubation with the secondary antibody at room temperature for 2 h. Visualization of protein bands was detected with ECL chemoluminescence staining detection kit (Bio-Rad) using densitometry of Bandscan 5.0 software that was used for quantifying the density of each protein band. With GAPDH as control, the expression of total protein was normalized.

### Statistical analyses

All statistical results are displayed as mean ± standard deviation and analyzed by Oneway analysis of variance (ANOVA) followed by Tukey’s post hoc test among groups using Statistical Product and Service Solutions (SPSS) (Version 17.0). *P* values less than 0.05 were considered as statistically significant difference between groups.

## Results

### Moracin increased cell viability in LPS-induced primary nucleus pulposus cells

**Figure 1:**
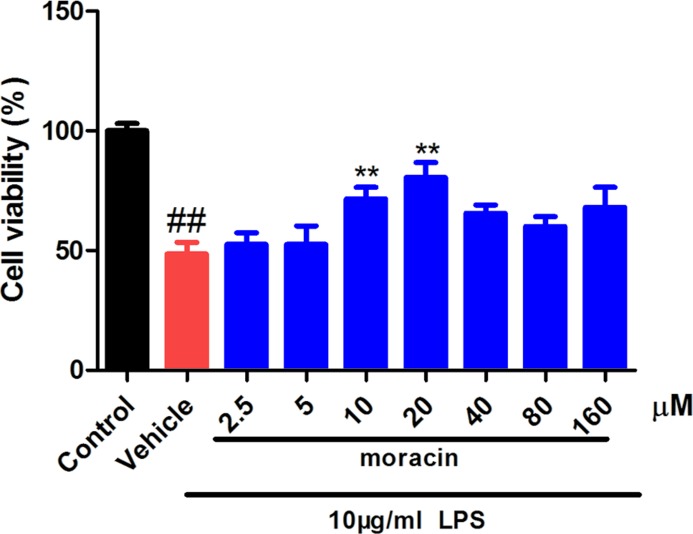
Effects of moracin on cell viability The moracin cell viability was measured by CCK8.The concentration of LPS with 10 μg/ml was used to induce the inflammation. The values are presented as the mean ± standard deviation. *P < 0.05 compared with the LPS group; **P < 0.01 compared with the LPS group; ##*P* < 0.01 compared with the control group.

In the first, it was determined that LPS significantly reduced the cell viability in primary nucleus pulposus cells. However, the cells treated with moracin at different concentrations (2.5, 5, 10, 20, 40, 80, 160 μM) significantly increased the cell viability, especially at concentrations of 5–20 μM (Figure 1). Thus, we closed moracin at 5, 10, 20 μM for the following experiments.

### Moracin reduced inflammatory cytokines in LPS-induced primary nucleus pulposus cells

**Figure 2:**
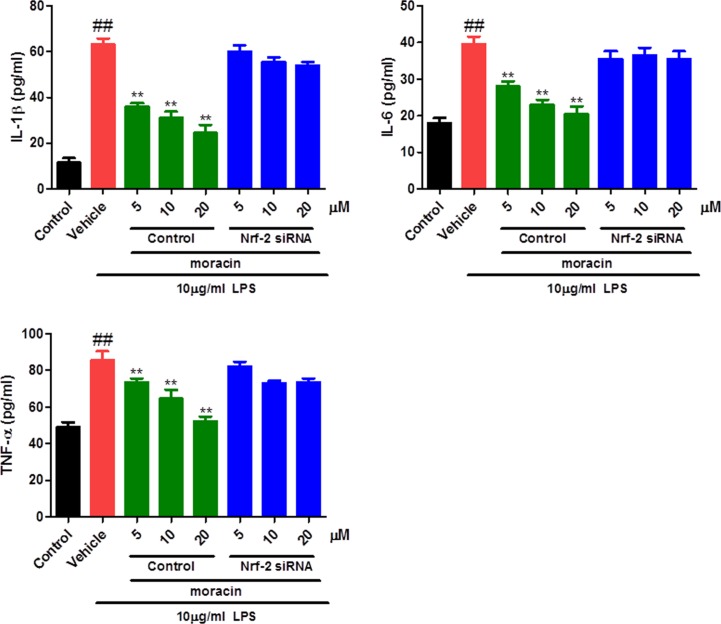
Effects of moracin on inflammatory cytokines in LPS-induced primary nucleus pulposus cells The concentrations of inflammatory cytokines IL-1β, IL-6 and TNF-α in cell supernatant were recorded by an enzyme-linked immunosorbent assay (ELISA) kits. The concentration of LPS with 10 μg/ml was used to induce the inflammation. The values are presented as the mean ± standard deviation. **P < 0.01 compared with the LPS group; ^##^*P* < 0.01 compared with the control group.

To explore the potential anti-inflammatory effects of moracin, the levels of IL-1β, IL-6 and TNF-α in the supernatant of primary cell cultures were measured by ELISA. As shown in our results, LPS challenge significantly increased the levels of IL-1β, IL-6 and TNF-α in comparison with that in control. As expected, moracin treatments markedly inhibited the production of inflammatory cytokines compared with that in the LPS group (Figure 2). Moreover, the anti-inflammatory effects of moracin were significantly reversed when treated with Nrf-2-specific siRNA.

### Moracin attenuated SOD, MDA and CAT in LPS-induced primary nucleus pulposus cells

**Figure 3:**
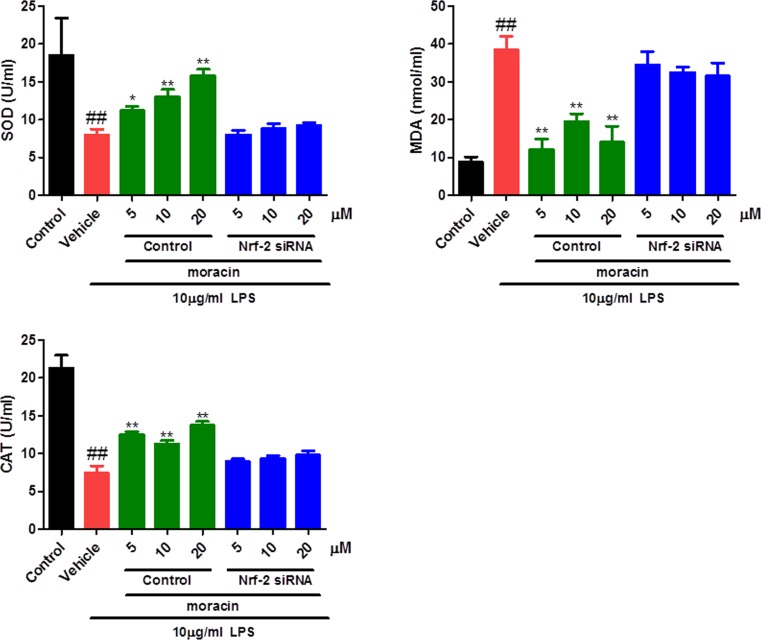
Effects of moracin on SOD, MDA and CAT in LPS-induced primary nucleus pulposus cells The levels of SOD, MDA and CAT in cell supernatant were recorded using the commercial kits on the basis of the manufacturer’s instruction. The concentration of LPS with 10 μg/ml was used to induce the inflammation. The values are presented as the mean ± standard deviation. *P < 0.05 compared with the LPS group; **P < 0.01 compared with the LPS group; ^##^*P* < 0.01 compared with the control group.

The antioxidant system was disturbed indirectly by the LPS challenge. As data shown in the present study, the nucleus pulposus cells treated with LPS exhibited reduced activities of SOD and CAT, and increased levels of MDA in the supernatant of primary cell cultures. As expected, moracin (5, 10, 20 μM) treatments remarkably increased the concentration of SOD and CAT, effectively decreased the vitalities of MDA. Also, the antioxidative effects of moracin were significantly reversed when treated with Nrf-2-specific siRNA (Figure 3).

### Moracin attenuated Nrf-2/HO-1 and NF-κB/TGF-β signaling in LPS-induced primary nucleus pulposus cells by western blot

**Figure 4:**
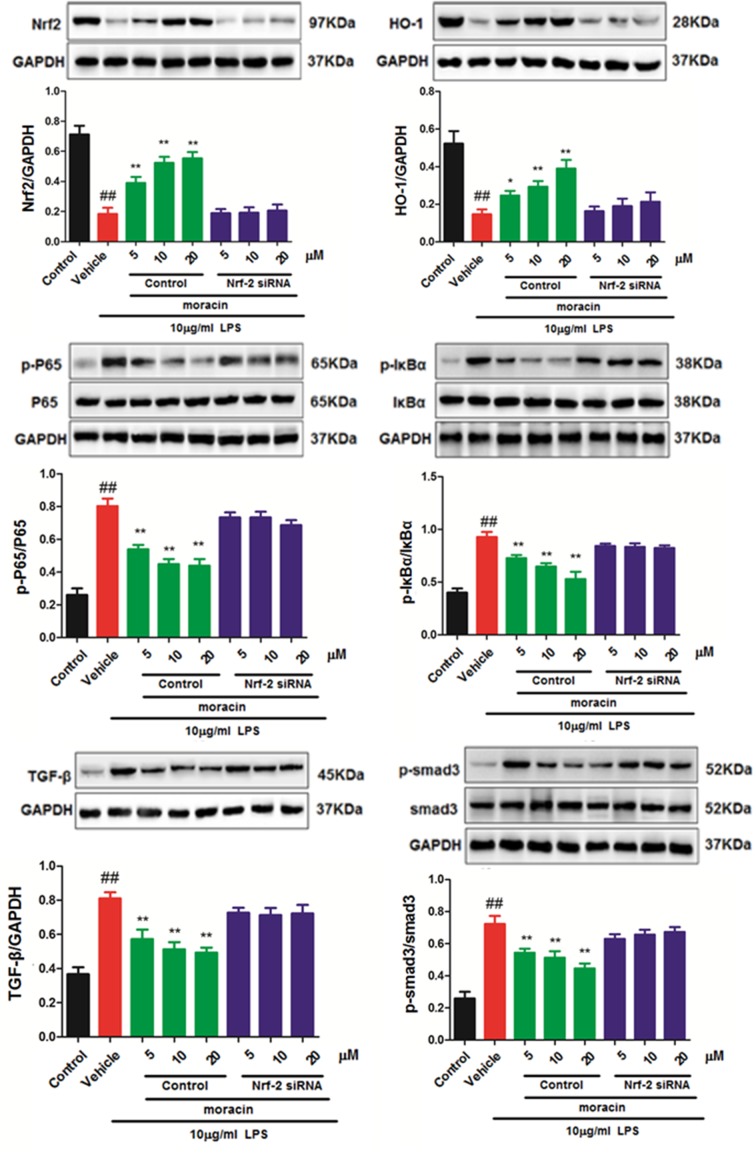
Effects of moracin on Nrf-2/HO-1/NF-κB/TGF-β signaling in LPS-induced primary nucleus pulposus cells The concentration of LPS with 10 μg/ml was used to induce the inflammation. The protein of Nrf-2/HO-1/NF-κB/TGF-β signaling in LPS-induced primary nucleus pulposus cells was detected by Western blot. The values are presented as the mean ± standard deviation. *P < 0.05 compared with the LPS group; **P < 0.01 compared with the LPS group; ^##^*P* < 0.01 compared with the control group.

To further investigate the possible mechanism of moracin on primary nucleus pulposus cells, the expressions of Nrf-2/HO-1 and NF-κB/TGF-β signaling were detected in LPS-induced primary nucleus pulposus cells. As shown in Figure 4, increased levels of p-NF-κBp65, p-IκBα, TGF-β and p-smad-3, and down-regulated levels of Nrf-2 and HO-1 were observed in LPS treatment model group compared with those in the control group. However, moracin (5, 10, 20 μM) treatments remarkably reverse those effects. Further, these benefit effects of moracin were obviously reversed when treated with Nrf-2-specific siRNA.

### Effects of moracin on MMP-3, MMP-13, Col-I, Col-II and aggrecan signaling/expression in LPS-induced primary nucleus pulposus cells by RT-PCR analysis

**Figure 5:**
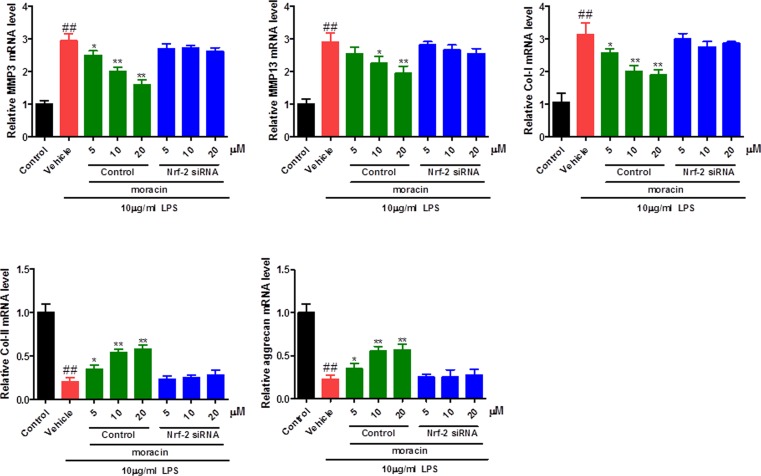
Effects of moracin on MMP-3, MMP-13, Col-I, Col-II and aggrecan signaling/expression in LPS-induced primary nucleus pulposus cells The concentration of LPS with 10 μg/ml was used to induce the inflammation. The expression of MMP-3 and MMP-13 in LPS-induced primary nucleus pulposus cells were detected by RT-PCR analysis. The values are presented as the mean ± standard deviation. *P < 0.05 compared with the LPS group; **P < 0.01 compared with the LPS group; ^##^*P* < 0.01 compared with the control group.

To further ensure the role of moracin on LPS-induced primary nucleus pulposus cells, the RNA levels of MMP-3, MMP-13, Col-I, Col-II and aggrecan were detected by PT-PCR analysis. An up-regulated mRNA expression of MMP-3, MMP-13, Col-I and a down-regulated mRNA expression of Col-II and aggrecan in LPS treatment model group compared with control group (Figure 5). In contrast, moracin (5, 10, 20 μM) treatments remarkably reverse those effects. Further, these benefit effects of moracin were significantly restored when treated with Nrf-2-specific siRNA (Figure 5).

## Discussion

The intervertebral disc works as shock absorbers during the loading of the spine. A damaged disc is hardly to self-repair and perform functions [10]. Intervertebral disc degeneration, which is thought as a significant cause of socio-economic problems, has been suggested as a common disease closely related to the inflammation of nucleus pulposus cells [11]. They mainly promote degeneration of the intervertebral disc by causing inflammatory reaction and inducing apoptosis, and various inflammatory factors can also affect each other. Targeted blocking the action of inflammatory factors in degenerative intervertebral discs, inhibiting the inflammatory response caused by it, and reducing the apoptosis of intervertebral disc cells, is of great significance for delaying the process of degeneration of intervertebral discs and reducing the clinical symptoms of patients [12]. Moracin is a biological active component obtained from the root barks of *Morus alba* L., which has been reported to exhibit anti-inflammation and antioxidant activities in different models [13]. However, few studies have reported its potential therapeutic effect on the degeneration of the intervertebral disc and its mechanism of action. In the present study, we demonstrated the ability of moracin to antagonize LPS-mediated inflammatory mediators in primary culture of nucleus pulposus including IL-1β, TNF-α and IL-6, suggesting a beneficial potential for its clinical application. Interestingly, the anti-inflammatory effects of moracin were significantly reversed when treated with Nrf-2 specific siRNA, indicating an important role for Nrf-2 targeting signaling. It is well known that the Nrf-2/HO-1 and NF-κB/TGF-β pathways act core roles in regulating inflammatory response [14,15]. Nrf2 is a redox-sensitive transcription factor, which promotes the transcription of antioxidants during the cellular redox state. Nrf2 interacts with cytoprotective factors HO-1 and SOD, and suppresses MDA and CAT to defense the oxidative stress damage. In our research, it was suggested its antioxidative effects of moracin, which significantly up-regulated the SOD and CAT activities, and effectively down-regulated the MDA activity. Western blot was used to evaluate the effects of moracin on the protein expression of Nrf-2/HO-1 signaling. Our results showed that moracin (5, 10, 20 μM) activated the Nrf-2/HO-1 signaling in a dosage-dependent manner.

Phosphoralaed smad2/3, associated with the common mediator smad4, translocates into the nucleus and activates the smad-dependent TGF-β signaling [16,17]. In addition, NF-κB signaling is important to dive cancer and inflammation via RNA [18–21]. Recently, NF-κB is also reported to play an important role in the intervertebral disc. In normal cells, the inactivated NF-κB is absorbed by the IκB protein in the cytoplasm. IKK complexes, IKKα and IKKβ, are the main regulator factors that can control the IκB promotion [22]. After the phosphorylation and degradation of IκB, NF-κB is immediately transferred to the nucleus that could drive the expressions of cytokines and inflammatory mediators [23,24]. After stimulated by LPS, NF-κB could transferred from cytosol to the nucleus, and then induced pro-inflammatory cytokine release, which will promote the activation of TGFβ signaling [25,26]. According to our results, after the treatment of moraxin (5, 10, 20 μM), the increased levels of p-NF-κBp65, p-IκBα, TGF-β and p-smad-3 induced by LPS were remarkably reversed. Thus, the NF-κB/TGF-β pathway was associated with the pharmaceutical effects of moracin in LPS-treated nucleus pulposus cells.

The up-regulated expression of MMPs was observed in intervertebral disc degeneration. According to previous studies [27,28], the expression of MMP-3 and MMP-13 were increased in degenerated human discs. As results shown in our study, increased levels of MMP-3 and MMP-13 induced by LPS treatment were also observed, and moracin (5, 10, 20 μM) treatments remarkably reversed these changes. Collagen-I, collagen-II and aggrecan are the main components of nucleus pulposus. The reduced content of collagen-II and aggrecan and increased level of collagen-I were observed in nucleus pulposus cells. As results shown in our study, increased level of collagen-I and decreased levels of collagen-II and aggrecan induced by LPS challenged were significantly reversed after moracin (5, 10, 20 μM) treatments.

In conclusion, the present study investigated the protective effects of moracin on LPS-challenged primary nucleus pulposus cells. Our results demonstrated the ability of moracin to antagonize LPS-mediated inflammation and oxidative stress via partly inhibiting of NF-κB/TGF-β pathway. These findings provided a potential candidate compound for the treatment of intervertebral disc degeneration. However, further study is still needed in the future.

## Abbreviations

CAT: catalase
ELISA: enzyme-linked immunosorbent assay
IL: interleukin
MDA: malondialdehyde
SOD: superoxide dismutase
TNF-α: tissue necrosis factor-α
